# The “state of the nation” in trauma critical care: Where are we?

**DOI:** 10.4103/0974-2700.41783

**Published:** 2008

**Authors:** Timothy C Hardcastle

**Affiliations:** Deputy Director Trauma Service, Inkosi Albert Luthuli Central Hospital Department of Health KZN, University of Kwazulu-Natal, Durban, South Africa. E-mail: timothyhar@ialch.co.za

## TRAUMA CRITICAL CARE: WHERE ARE WE?

Trauma is the direct result of man's interaction with himself, his neighbor, and the environment. The challenge in the care of the trauma patient is that the groups of persons who largely fall into the trauma population are the young previously healthy and potentially economically productive age ranges.[1]

The treatment of the trauma patient has progressed over the past 40 years since the introduction of the Advanced Trauma Life Support® course of the American College of Surgeons,[2] through the development of similar courses for nursing such as the Trauma Nursing Core Course[3] and Pre-hospital providers (Pre-hospital Trauma Life Support),[4] and then recently the introduction of the Definitive Surgical Trauma Care™[5] course of the International Association for Trauma Surgery and Intensive Care (IATSIC), a grouping within the International Society of Surgery (ISS-SIC) based in Switzerland. Education of trauma providers is thus available from initial assessment through to definitive care. Postspecialization Trauma Fellowships are available in a number of countries on both sides of the equator.

The challenge to trauma care, however, remains in countries where the trauma load outstrips the facilities and this too has recently been addressed by the World Health Organization through the Primary Trauma Care project, with the publication of a set of standards to be aimed for by developing nations.[6]

Trauma Critical Care takes this paradigm a step further still, attempting to address the needs of the most severely injured patients beyond the emergency department through to the time where rehabilitation can occur. It calls for the availability of skilled personnel with the correct caregiver: patient ratios, sophisticated equipment and the associated sundries, and support services. This is a great challenge to developing nations where the major challenges in healthcare include infectious disease and poor hygiene rather than the diseases of aging and lifestyle.

## SO WHERE ARE WE IN THE FIELD OF TRAUMA CRITICAL CARE?

Aspects of initial care and the impact on the critically ill patient: Prevention and precaution is better than reaction.

It has been recognized that the three major reasons why trauma patients die after the initial phase of care is due to acidosis, hypothermia, and coagulopathy - these due to the loss of vital circulation volume and certain, as yet, incompletely understood, biochemical, and hormonal reactions. I propose that the underlying basis for all of the results of trauma can be laid at the feet of the 3H's: Hypoxia, Hypoperfusion, and resultant Hypothermia. These three factors have been known to cause the second hit phenomenon in the brain-injured group[7] and can certainly take the blame for outcome after other trauma too; hypoxia causing the acidosis due to anaerobic metabolism, aided by the hypoperfusion, which also aids induction of the hypothermia. The hypoperfusion increases the acidosis and the hypothermia inducing the coagulopathy. Coagulopathy is, however, not primarily related to the consumption of coagulation factors, as demonstrated by Brohi and coworkers recently, rather it is a component of the complex and poorly understood inflammatory response to trauma.[8] So the lethal triad leads to a “bloody vicious cycle” spiraling down to rapid death.[9]

A study in 1994 showed that some patients with penetrating truncal wounds had reduced mortality when given less fluid therapy and rapid surgical intervention.[10] This concept has been extensively explored through animal studies and human trials over the past few years and the majority of these trials indicate that there is a certain group of patients who benefit from such a strategy. This has now been termed permissive hypotension.[11] The group of patients who would benefit from this strategy includes adolescents and adults with noncompressible bleeding where normotension would increase the bleeding and the resultant coagulopathy. Exclusions include patients such as the pregnant patient, the elderly and small children where any hypotension increases the risk of hypoperfusion to the brain or foetus as the case may be or where prolonged entrapment places the patient at risk for myoglobinuria. The concept has been further investigated through the use of limited volume resuscitation and particularly with the use of hypertonic saline, either alone or with a diluent solution (dextran or hydroxyethylstarch).[12] While there is no absolutely conclusive evidence for or against the hypertonic fluid concept, it has been adapted for use in pre-hospital care in the austere environment of war. Indeed the outcomes appear, at first glance, to be at least equivalent to current fluid therapy regimens.

Early definitive surgical care and the role of the Damage Control concept: Emanating from the proof of improved outcome from early surgical intervention is the concept of abbreviated surgery and later definitive reconstruction of the severely injured trauma patient, initially starting in the abdomen and now extending to the chest, neck, vascular injury, and orthopedics. This “Damage Control” concept brings patients earlier to the Trauma intensive care unit (ICU), often with the patient requiring extensive ongoing resuscitation and with the realization of the need to possibly undertake early or later definitive surgery.[13]

Damage control orthopedics is the extension of the philosophy of abbreviated care to the orthopedic injury spectrum. Early definitive fixation of certain long bone fractures has been shown to decrease the risk of Fat Embolism Syndrome,[14] while the use of fracture stabilization with external fixators has been shown to achieve the same result, often within a shorter time span. Selection of the appropriate patient for definitive early “total care” or temporizing external fixation is the area of extensive ongoing research in orthopedics today.[15]

## HAVE THESE STRATEGIES IMPROVED OUTCOME?

Recent data suggests that there is indeed an improved overall survival[16] with more patients who have higher ISS scores and are, therefore, more ill surviving to admission to the ICU, thus predicting longer length of stay and more potential for complications.

This begs the question: What are we able to offer today in the Trauma ICU that has contributed to the improved survival of these more severely injured patients and also, where is the limit of our ability to provide care that does not just prolong suffering?

### In the ICU

#### Lung protective ventilation strategies for patients with blunt chest trauma

The recently completed acute respiratory distress syndrome -network trial[17] has revolutionized the way that critically ill patients are ventilated and together with the clearer definitions of acute lung injury and Acute Respiratory Distress Syndrome[18] have enabled the Intensivist to target ventilation strategies to the needs of the patient, decreasing the complication rates, and improving survival. This has been applicable in the case of the trauma patient also, with the lower tidal volume, faster rates, controlled peak and plateau pressures, and appropriate use of Positive End Expiratory Pressure resulting in greater numbers of survivors and increased ventilator-free days.

#### Sepsis, Systemic Inflammatory Response Syndrome (SIRS), septic shock, and the trauma patient

The trauma patient presents a challenge to the Intensivist from the point of nonconformity to the usual criteria used to classify the severity of the physiological response to the injury.[19] The reason for this is that traumatic shock is often “sterile,” i.e. there is no true evidence that there is an “infective source” as the contributing factor for the presence of the high temperatures, raised inflammatory markers and hypotension, especially in the context of the patient in whom one of the modern resuscitation strategies has been utilized. This is especially true of the trauma patient in the ICU who has presented acutely after injury and where no obvious “missed injuries” with a septic source are identified. This calls for a review of the definitions originally proposed to classify SIRS, Sepsis, and Septic shock[20] to account for this “sterile” shock picture, importantly to avoid the use of broad-spectrum antibiotics unnecessarily in this patient group.

#### Rational antibiotic use and the prevention of resistance

Following on from the previous point, there is the need for constant vigilance in our ICU's against induction of antibiotic resistance leading to the so-called “superbugs.”[21] Much research is still required into effective prophylaxis and therapy protocols tailored to the trauma victim. Some of these issues have been addressed by the Eastern Association for the Surgery of Trauma-group (www.east.org) through the development of practice guidelines for antibiotic use, but local resistance patterns must still determine local policy. This is a good area for translational research in trauma, especially from the Third World where these patterns are poorly documented.

#### Glucose control and the role of steroids

The work of the Van Der Berghe group[22] initially fueled enthusiasm for tight glucose control in all critically ill patients and this was rapidly extended to the trauma patient, despite there being very few trauma patients in the original study. Subsequent publications in the trauma subgroup[23,24] seem to suggest that outcome is identical whether the principle of tight control (4.5-6 mmol/l [80-110 mg/dl]) is followed, or when the glucose is kept in the range between 6 and 8 mmol/l [110–150 mg/dl]). The latter method seems to have less hypoglycemic episodes, which may be more detrimental than hyperglycemia.

#### The Etomidate saga and the major trauma patient.

Annane[25] proposed Etomidate as an independent factor in the mortality of septic shock, through the suppression of the adrenal gland function of 11-beta-hydroxilase, even after a single bolus dose during the resuscitation phase. This was particularly evident in the group of patients where low dose corticosteroids were not utilized, although conclusive evidence for an increase in mortality were lacking. However, the cause-effect relationship is difficult to determine and the argument may be upheld that the patients in whom Etomidate was used had a worse clinical picture thus prompting the use of the more cardio-stable drug compared to the other induction agents studied. Again, few trauma patients were included his cohort. To date, only one small prospective study in Trauma patients in the ICU has been presented at a recent congress[26] and the data could well be flawed by the small numbers in the study and confounding factors. One retrospective cohort study[27] in trauma patients revealed the presence of nonresponse to a cosyntropin stimulation test was associated with the administration of Etomidate. The cause-effect relationship could not be conclusively confirmed as this same patient group had a higher number of patients with hemorrhagic shock compared to responders. This may imply use of Etomidate is a reflection of the underlying pathology, which of itself may lead to adrenal suppression, rather than Etomidate being the sole cause, although this is an unlikely scenario. Recent work on the role of steroid supplementation[28] has questioned Annane's original conclusions of the role of steroids in septic shock and this may lead to the only real indication for physiological steroid supplementation being the use of Etomidate for rapid sequence intubation. The relative risk to benefit of the use of a stable agent compared to other induction agents may, however, still make Etomidate the agent of choice. This is indeed an area in need of good quality prospective randomized study to determine whether there is indeed a causal relationship rather than just an association.

#### Renal replacement therapy (RRT) - mode and timing

Renal impairment has been clearly shown to be a major determinant of a poorer outcome in the ICU,[29] but there remains much debate as to the optimal timing of the initialization and type of RRT. In the trauma subgroup, there is often the additional effect of the pigment-related myoglobinopathies which carry a better overall prognosis, provided these are dealt with early. The publication of the risk, injury, failure, loss, and end-stage Kidney definitions[30] has enabled rational early RRT to be implemented and allows for comparable studies to determine the point of maximal benefit in the trauma subgroup. Currently the standard of care appears to be continuous venovenous hemodialysis (CVVHD) or sustained low efficiency dialysis (SLED). The lack of availability of dialysis services in many Third World countries serves as an additional incentive to prevent renal dysfunction.

#### Thromboprophylaxis and stress ulcer prophylaxis

Deep venous thrombosis and the resultant pulmonary emboli have been dramatically reduced by the aggressive use of thromboprophylaxis, while the incidence of stress ulceration has dramatically been reduced in the ICU by the modern strategies of early enteral feeding and better overall resuscitation. However, clinical compliance with the overwhelming evidence is lacking in many places when audits of care are performed and this remains an ongoing challenge to the providers of critical care.[31] Better compliance is required for an ongoing improvement in outcome.

#### *Outcome prediction* - no reliable scores specifically for trauma, particularly for penetrating trauma.

Many different scoring systems have been devised for the prediction of outcome of the trauma patient, with the Sequential Organ Failure Score holding the most promise currently for outcome prediction in the ICU.[32] Failure of four or more organs is universally associated with an almost 100% mortality. The problem with many of the trauma-specific scores, including the Injury Severity score[33] is that they make little allocation for the multiple injured single region, while often weighting heavily toward the head injured and blunt injured patient groups. This area of Trauma Critical Care is a specific one for careful translational research to develop reliable, repeatable, and yet, simple scoring systems to accurately predict outcome after critical injury.

#### The concept of futile care and the withholding or withdrawing of therapy

There comes a time in every person's life when the hand of modern medicine cannot, or possibly should not, intervene and prolong suffering. In the modern era of self-determination one needs to respect patient's wishes to not have unnecessary life-prolonging therapy instituted. It is of paramount importance that all societies look to developing acceptable systems of advanced directives to prevent undue suffering and heartache to families. Additionally, there comes a time when clinicians need to advise family against the persistence of therapy at all costs in a fairly hopeless scenario. There is no legal, moral, or ethical responsibility upon any clinician to continue with a therapy that is determined to be futile and clinicians need to be mature and courageous enough to share these concerns with the family of the dying. Additionally clinicians need to derive policies and procedures for the capping (withholding of additional therapy) or withdrawal of noncontributory therapy to prevent the prolongation of the inevitable outcome. This does not stop us from providing care - analgesia, sedation, anxiolysis, and an open airway should always be assured. Guidelines exist in the critical care literature for the guidance of the healthcare professional in these matters.[34]

## CONCLUSION

There are many challenges to the treatment of the severely injured and many of the areas will need further research to effectively guide us as clinicians in the future. Hopefully this fairly brief overview has highlighted some areas for research, has challenged readers to an evidence-based approach to the critically ill and will lead to the submission of many papers in this field by researchers across the spectrum from bench-research to clinical patient care, enabling us to better treat our patients in the future through the translation of research into practice.
